# The Radcliffe Infirmary, Oxford: Its Re-Creation Completed

**Published:** 1913-12-06

**Authors:** 


### THE RADCLIFFE INFIRMARY, OXFORD.

### Its Re-creation Completed.

It has taken quite twenty-five years to quicken public opinion, to provide the necessary funds and to change a hospital, which was obsolete up to 1891, into a modern hospital containing several units of value and interest. Reconstruction rather than rebuilding has mainly characterised the work, which was completed on November 26, 1913, with the formal opening of the new buildings by the Chancellor, Lord Curzon. This new wing does infinite credit to the architect, Mr. Edward Warren, and to the technical experts who have been associated with him, several of whom will be active members of the staff. The latter gain the privilege of working in the new departments in this building. They have been carefully selected, are men of marked ability, and we look forward with confidence to the successful organisation of a Graduate School. This should become a centre for consultations of the utmost efficiency and interest, for here should come every practitioner of merit working within a radius of twenty miles from the Radcliffe Infirmary. Sir William Osler was the first man to suggest a new departure of this kind on the part of every county hospital in England and Wales. It is fitting that he should be the first to be provided with the fullest facilities, as Regius Professor of Medicine at Oxford, to enable him to elaborate and perfect the first of such graduate schools and centres of consultation, in connection with a great and important county hospital. We published the plans of this new wing in The Hospital,

February 3, pp. 459, 460, and a letter from Mr. Warren thereon in The Hospital, February 17, 1912. The actual results achieved by the completed reconstruction of the Radcliffe Infirmary may well form interesting matter for an article on a future occasion.

The speakers at the meeting on 26th ult. did honour to the memory and services rendered to the infirmary by good friends of that institution including the founder, Dr. John Radcliffe, with whom the Chancellor coupled the name of Mr. John Briscoe, as the latest benefactor. Lord Curzon made some good points and mentioned that it was 250 years since Dr. Radcliffe came as an undergraduate to Oxford University. Sir William Osler, speaking of John Radcliffe, declared that he belonged to the interesting crowd of physicians whose names had gone down to posterity associated with the great benefactions they had been able to give through proper dispensation of their wealth. "Radcliffe," declared Sir William Osler, "was an extraordinary personality; he was not remembered as a physician; he gave nothing practically to literature; he was remembered by great benefactions and the stories still associated with his name." When Radcliffe started practice he had twenty remedies for every disease he met with: when he retired from practice he declared he had twenty diseases for which he had not a single remedy. Radcliffe bequeathed large funds to Oxford University, which have ever since been administered by trustees. The number and importance of the services rendered to the University by the outlay of the money derived from Dr. Radcliffe's Trust were strikingly brought out in the speech of Lord Curzon. This article is only concerned with one of them, the foundation known as the Radcliffe Infirmary. Lord Curzon did well to emphasise the fruitfulness and value of Radcliffe's generosity, because at the present day, as the Chancellor said, "Study is often a precarious interlude between a series of paroxysms." Even the members of a great university, like people outside, are apt to forget what deceased benefactors have done for them.

The names mentioned of former treasurers, who had done yeoman service, were: The late Dr. Bright, Rev. Mr. Fletcher, Lord Dillon, and other old veterans in the service of the hospital, like Dr. Gray and Mr. Whitfield. Mr. H. B. Symonds gave an amusing account of John Briscoe, F.R.C.S., who became house surgeon to the Radcliffe Infirmary in 1845. He found that its condition was unsatisfactory, and he set to work and carried out many great improvements. Mr. Symonds described Briscoe as a skilful surgeon who was materially aided by an excellent matron, and so great improvements took place in the nursing. Briscoe was a supporter of Lister's methods, which he encouraged in every possible manner. He was surgeon to the Radcliffe until 1881, and never lost his interest in the hospital, where he was accustomed to attend operations and go round the wards. When he was permanently confined to his bed, he had a list of the operations performed brought to him regularly, and three hours before he died he asked the nature of the operations performed at the infirmary on that day! John Briscoe left £63,000 to the hospital, to the surprise of his contemporaries, who had no idea of his wealth. This fact, taken in conjunction with a recent incident in connection with the Royal Free Hospital, enforces once more the sound principle that every visitor to a voluntary hospital should invariably be treated with extreme courtesy.

### Present Benefactors and Staff.

The public have altogether contributed £9,000 towards a total expenditure of £25,000. Once again the dead hand has therefore beaten the living, a fact which cannot too often be driven home to the minds and consciences of men and women now alive, who benefit, directly or indirectly, all the time by the work of the voluntary hospitals. We regret we have not space to print Lord Curzon's speech in full. It was both witty and able and should be read by all who take an interest in or have to speak in support of a hospital or medical institution. It is a remarkable fact, highly gratifying to the present Chancellor of Oxford University, that in 1893 Lord Salisbury, who was then Chancellor, speaking on behalf of the Radcliffe Infirmary in the Sheldonian Theatre, declared: "Its proper position is to be equipped with all that science and practice can furnish to make it the most efficacious means for bringing medical knowledge to bear on the relief of the suffering of our kind." Twenty years afterwards the present Chancellor, Lord Curzon, has opened a new pathological and clinical department with its up-to-date casualty theatre and adjuncts, being the last of the group of buildings, the completion of which places the Radcliffe Infirmary in the position which it was the late Lord Salisbury's hope that it would one day occupy. That this should be so is a striking proof of the excellence and value of the work done by all who are at present responsible for the administration of this institution.

Mr. Symonds paid a deserved tribute to the present resident staff. He pointed out that in 1845, in Mr. Briscoe's day, there were nine day nurses and one night nurse with 100 beds; in 1913 there are 42 day nurses and 10 night nurses and upwards of 160 beds. In Mr. Briscoe's time no anæsthetics were administered to patients at the hospital, but in 1913 anæsthetics were administered 2,089 times. The Matron and Secretary were indefatigable in their attention to the guests, and must have had a good deal of anxious work in the way of preparation for the entertainment of so large a company.

The Radcliffe Infirmary by shaping its course with wisdom secures the promise of extended progress and good fortune. The fact that it is at the present time under the direction of the Rev. G. B. Cronshaw, an able and deservedly popular treasurer, affords the best guarantee to the governors and to the University of Oxford that the Radcliffe Infirmary will continue to advance as the years pass.

